# Missed opportunities to deliver intermittent preventive treatment for malaria to pregnant women 2003–2013: a systematic analysis of 58 household surveys in sub-Saharan Africa

**DOI:** 10.1186/s12936-015-1033-4

**Published:** 2015-12-23

**Authors:** Kathryn G. Andrews, Michael Lynch, Erin Eckert, Julie Gutman

**Affiliations:** Harvard T. H. Chan School of Public Health, Boston, MA USA; Malaria Branch, Centers for Disease Control and Prevention, Atlanta, GA USA; Global Malaria Programme, World Health Organization, Geneva, Switzerland; President’s Malaria Initiative, USAID, Washington, DC USA

## Abstract

**Background:**

Despite the availability of effective preventive measures, including intermittent preventive treatment for malaria during pregnancy (IPTp), malaria continues to cause substantial disease burden among pregnant women in malaria-endemic areas. IPTp coverage remains low, despite high antenatal care (ANC) attendance. To highlight areas of potential improvement, trends in IPTp coverage were assessed over time, missed opportunities to deliver IPTp at ANC were quantified, and delivery of IPTp was compared to that of tetanus toxoid (TT).

**Methods:**

Data from 58 Demographic and Health Surveys conducted between 2003 and 2013 in 31 sub-Saharan African countries, with relevant questions on IPTp, ANC and TT were used to assess ANC attendance, and IPTp and TT delivery. A missed opportunity for IPTp delivery is an ANC visit at which IPTp could have been delivered according to policy but was not.

**Results:**

The proportion of pregnant women who received ≥2 doses of IPTp increased in surveyed countries from nearly zero before to a median of 29.6 % (IQR 20.1–42.5 %) seven or more years after IPTp policy adoption. ANC attendance was high (median 76.6 % reported ≥3 visits); however, even seven or more years post policy adoption, a median of 72.9 % (IQR 58.4–79.5 %) ANC visits were missed opportunities to deliver IPTp. Among primigravid women, a median of 61.5 % (IQR 50.9–72.9 %) received two doses of TT; delivery of recommended TT exceeded IPTp in all but one surveyed country.

**Conclusions:**

IPTp coverage measured by household surveys is unsatisfactorily low, even many years after policy adoption. The many missed opportunities to deliver IPTp suggest that deficiencies in delivery at ANC are a significant contributing factor to the low coverage levels. High levels of TT delivery indicate capacity to deliver preventive measures at ANC. Further research is required to determine the factors driving the discrepancies between IPTp and TT coverage, and how these may be addressed to improve IPTp coverage.

**Electronic supplementary material:**

The online version of this article (doi:10.1186/s12936-015-1033-4) contains supplementary material, which is available to authorized users.

## Background

Despite international calls for malaria eradication [1] and the availability of many low-cost interventions to prevent malaria morbidity and mortality, the disease continues to be responsible for a substantial public health burden in affected populations, causing one death every minute [2]. In 2013, there were an estimated 198 million (uncertainty range 124–283 million) malaria cases, and 584,000 (uncertainty range 367–755,000) malaria deaths globally, the majority of which occurred in sub-Saharan Africa, primarily among children under 5 years old [3]. In addition to children under five, malaria disproportionately affects pregnant women, and an estimated 125 million pregnancies occur annually in malaria-endemic areas [4]. Malaria in pregnancy is associated with increased risk of maternal anaemia, low birth weight, and neonatal mortality [5].

Along with insecticide-treated bed nets (ITNs) and effective treatment and diagnosis of clinical malaria, intermittent preventive treatment for malaria during pregnancy (IPTp) with sulfadoxine–pyrimethamine (SP) is one of the strategies recommended to prevent the adverse consequences of malaria in pregnancy [6]. IPTp consists of administering a single-dose, oral anti-malarial to all pregnant women, irrespective of whether they have malaria. Currently, SP is the only drug recommended for IPTp. IPTp-SP reduces maternal malaria and anaemia and improves infant birth weight [7]. The effect on birth weight is maintained even in the context of widespread ITN use and in areas with widespread resistance to SP [5]. In an analysis of national survey data, IPTp-SP reduced the odds of low birth weight (adjusted odds ratio 0.75 [95 % confidence interval CI 0.71–0.80]) compared to not using either IPTp-SP or ITNs; the protective effect remained even for women who lived in households with an ITN [8]. It is a highly cost-effective intervention for preventing maternal malaria and reducing neonatal mortality [9]. Since 2004, the World Health Organization (WHO) has recommended that women receive a minimum of two doses of IPTp-SP during pregnancy. In 2012 the policy was updated to recommend that IPTp-SP be administered at every scheduled antenatal care (ANC) visit starting in the second trimester, provided that doses are at least 1 month apart [6]. As WHO recommends three ANC visits during the second and third trimesters of pregnancy [10, 11], there should be ample opportunities for administration to allow a high proportion of women to receive three doses. Since 2011, the goal set by Roll Back Malaria has been that by 2015, 100 % of pregnant women at risk of malaria should receive at least two doses of IPTp-SP, in settings where IPTp is appropriate [12]. Despite relatively high ANC attendance, and the modest system requirements needed to deliver a single-dose, oral prophylactic in a clinic setting, coverage of IPTp-SP has been low in sub-Saharan Africa [13].

Coverage of health interventions delivered at a health facility depends on attendance of targeted groups and delivery of the intervention at a facility. For IPTp-SP, pregnant women may present too late or too few times for ANC to receive the recommended number of doses; however, some studies suggest that healthcare workers do not administer SP, either due to confusion over the guidance or because they are too busy or lack supplies [14]. Quantifying the missed opportunities to deliver an intervention allows for assessment of the effectiveness of the healthcare system to deliver that intervention, isolating the effects of shortcomings in provision of interventions at point-of-care from patients’ failure to seek care. Following the most recent WHO guidelines for administering IPTp-SP [10], for women not taking cotrimoxazole prophylaxis, each scheduled ANC visit in the second or third trimester is an opportunity to deliver IPTp-SP, and any of these visits where IPTp-SP is not administered could be considered a ‘missed opportunity’.

Many interventions are delivered to women through the ANC platform. Tetanus toxoid immunization (TT) has been routinely administered to pregnant women since the 1980s to prevent neonatal tetanus, an acute disease which generally presents in the first 2 weeks of life and carries a 10–100 % case fatality rate, depending on the level of care received [15]. Two doses of tetanus toxoid (TT2) delivered at least 4 weeks apart (at the first and second ANC visits) are recommended for all primigravid women. In contrast to IPTp-SP, TT2 routinely achieves high coverage levels [16], despite the fact that it is an injection and requires refrigeration [17]. To understand how successfully health systems are delivering IPTp-SP, all available surveys from the Demographic and Health Surveys (DHS) programme [18] from 2003 to 2013, spanning the implementation of IPTp-SP programmes, were used to explore missed opportunities for IPTp-SP administration and compare IPTp-SP coverage to that of TT.

## Methods

### Data

**Fig. 1:**
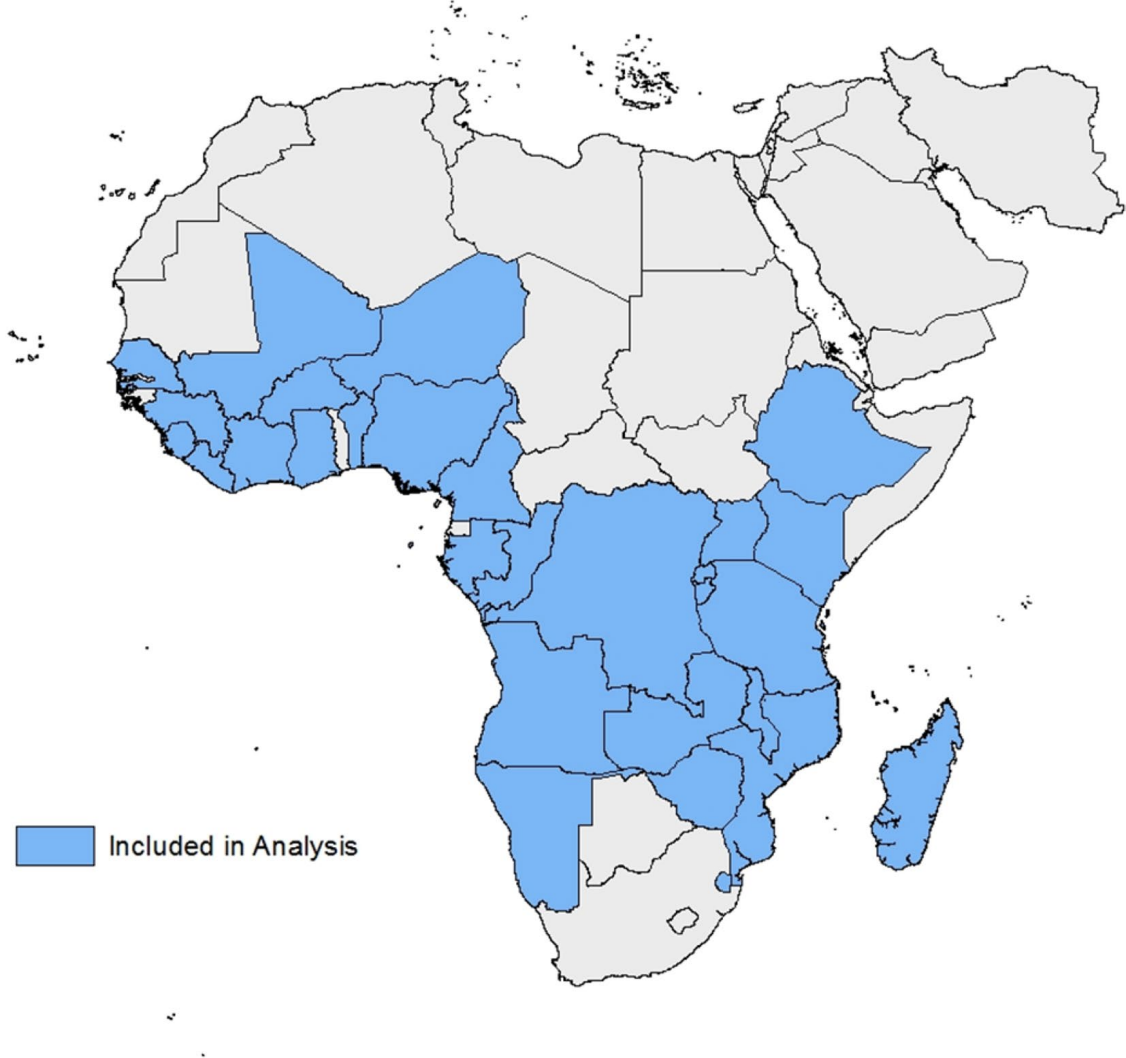
Map of Africa showing countries included in the analysis. A total of 58 surveys conducted by Measure DHS between 2003 and 2013 were included from the 31 *shaded* sub-Saharan African countries [18]

Seventy household survey datasets from the DHS programme website [18] conducted between 2000 and 2013, with relevant survey questions, were identified; these included standard DHS, AIDS indicator surveys (AIS), and malaria indicator surveys (MIS) from 32 countries in sub-Saharan Africa (Fig. 1; see also Additional file 1: Table S1 for a complete table of included surveys and respective IPTp and TT coverage). Twelve surveys where information on precise numbers of IPTp-SP doses was missing were excluded, leaving 58 surveys from 31 countries (2003–2013) for analysis. All of these surveys included a module with questions on pregnancies resulting in live births in the past 5 years and relied on women’s ability to recall their pregnancy experiences over this time period [18]. To ensure that estimates were calculated using independent observations from recent births, analyses were performed using, for each woman surveyed, the most recent pregnancy resulting in a live birth within the 2 years prior to the survey date. Data on ANC attendance and TT administration were available in 44 of the 58 surveys. STATA SE statistical software package version 13.1 (StataCorp, College Station, TX, USA) was used for all analyses, and all mean and CI estimates were weighted to account for survey design.

### Data analysis

Among women whose most recent pregnancy resulted in a live birth in the past 2 years, the proportion receiving two or more doses and the proportion receiving three or more doses of IPTp-SP were calculated for each survey. As nearly all surveys were conducted before the updated WHO policy in 2012 emphasizing dosing of IPTp-SP at each ANC visit, the proportion receiving two or more doses of IPTp-SP was used as the primary comparison in this analysis. In addition, to assess implementation of IPTp-SP over time since policy adoption, the median coverage across countries was assessed for surveys conducted during three periods in reference to IPTp-SP policy adoption: (1) pre-adoption, which included any surveys conducted from 2003 until the year after policy was adopted in a given country; (2) early post-adoption defined as the 5 year period starting 2 years after the policy was adopted; and (3) late post-adoption, which included any surveys conducted seven or more years after a country formally adopted an IPTp-SP policy (see Additional file 1: Table S1 for a list of surveys and their policy periods). Year of IPTp policy adoption was taken from the World Malaria Report and proposals that were submitted to the Global Fund to Fight AIDS, Tuberculosis and Malaria [19]. Because IPTp-SP coverage calculated from a given survey was actually based on data from the 2 years preceding the survey, and a survey was not available in every year for each country, presenting results based on these groupings (rather than calendar year) allowed for more meaningful interpretation of changes in coverage. In addition, this grouping allowed for lag time between adoption and implementation of a new policy. For those countries where two or more surveys were available, with at least one following the adoption of an IPTp-SP policy, the median 5 year annualized difference in IPTp-SP coverage was calculated between the surveys by taking the absolute difference in percentage points of coverage between one survey and the next within the same country and dividing this difference by the number of years that elapsed between the surveys. Since the annual change was quite small, it was multiplied by five to provide a more salient value, which is interpreted as the change in coverage over 5 years. This metric provides an estimate of the absolute change in coverage over time.

Among women whose most recent pregnancy resulted in a live birth in the past 2 years, the percentage of missed opportunities to deliver IPTp-SP was defined as:

$$\frac{{\# \,ANC \,visits \,in \,2nd \,and \,3rd \,trimesters\,\text{ - }\,\# \,IPTp \,doses \,given \,during \,these \,visits}}{\# \,ANC \,visits \,in \,2nd \,and \,3rd\, trimesters}$$

Given the limitations of available data, some assumptions were required for this calculation. As only the timing of the first visit, but not of subsequent visits, was collected in these surveys, it was assumed that no more than one first trimester visit occurred (WHO recommends only one visit in the first trimester [11]). In other words, the number of ANC visits occurring in the second and third trimester was calculated by subtracting one visit from the total number of ANC visits for any woman who reported that her first visit occurred in the first trimester. All ANC visits were assumed to take place at intervals of at least 1 month and all IPTp-SP doses reported were assumed to have occurred during ANC visits. HIV-infected women taking cotrimoxazole prophylaxis for the prevention of opportunistic infections are not eligible for IPTp-SP because of the increased risk of adverse drug reactions when these medications are taken concomitantly [20], and therefore their ANC visits should not be included in the calculation of missed opportunities. However, in the household surveys analysed, no data were collected on cotrimoxazole administration, therefore it was not possible to account for this.

To understand how coverage of other interventions delivered through ANC compares with that of two or more doses of IPTp-SP (IPTp-SP2+), the coverage of two or more doses of TT (TT2+) among primigravid women was compared to the coverage of IPTp-SP2+ among the same group of women. TT2+ coverage during pregnancy was calculated as the percentage of primigravid women in the two years prior to the survey who reported receiving at least two doses of TT.

The median, interquartile range (IQR), minimum and maximum across all survey estimates for the pre, early-post, and late-post policy adoption periods are presented to provide insight into the central tendencies of the estimates of interest. Although it is informative to present statistics across groups of surveys, it is important to stress that each of the surveys produces mean estimates that are only truly representative of the country and time period in which they are conducted, and the median value of the survey means cannot be interpreted, for example, as the prevalence across all of sub-Saharan Africa.

## Results

### Antenatal care

Data from 172,506 women across 44 surveys were used to assess ANC attendance. Overall, ANC attendance was high across surveys, with a median value of 92.7 % (IQR 87.4–95.6 %), 88.2 % (IQR 81.9–92.3 %), 76.1 % (IQR 67.5–81.6 %), and 48.6 % (IQR 41.7–59.6 %) of women attending at least one, two, three, and four visits, respectively. Only a small proportion of women (median of 7.2 %, IQR 3.6–19.7 %) attended ANC in the first trimester. The first visit occurred at median gestational age of 4.6 months (IQR 3.9–4.9). There was an increase in prevalence of two or more ANC visits from a median of 83.5 % in the pre-policy period to 89.2 % in the early post-adoption phase, staying constant at 89.0 % in the late post- adoption period. The prevalence of three or more ANC visits varied little, from a median of 73.6 % in the pre-adoption period to 75.6 and 76.6 % in the early and late post-adoption periods, respectively.

### Preventive treatment for malaria during pregnancy with sulfadoxine–pyrimethamine

Data from 205,200 women surveyed whose most recent birth was in the 2 years prior to the survey date were used to assess trends in IPTp-SP coverage over time. IPTp-SP became policy in 2004–2005 in 16 of the 31 countries in this analysis, and by the end of 2006 it was policy in 28 countries (Additional file 1: Table S1). Ethiopia, Burundi and Swaziland never implemented IPTp-SP, and Rwanda implemented IPTp-SP only from 2005 to 2008 and then abandoned the policy. Prior to the existence of a policy in country, IPTp-SP2+ coverage was nearly 0 (median of 1.1 % based on 15 pre-adoption surveys occurring 2003–2012), as expected. Coverage of IPTp-SP2+ improved to a median of 20.2 % (IQR 12.9–40.3 %) in the 25 surveys conducted during 2003–2011 in the first 7 years following adoption of IPTp-SP as policy, and to a median of 29.6 % (IQR 20.1–42.5 %) in those 18 surveys conducted during 2004–2013 more than 7 years following policy adoption.

**Fig. 2:**
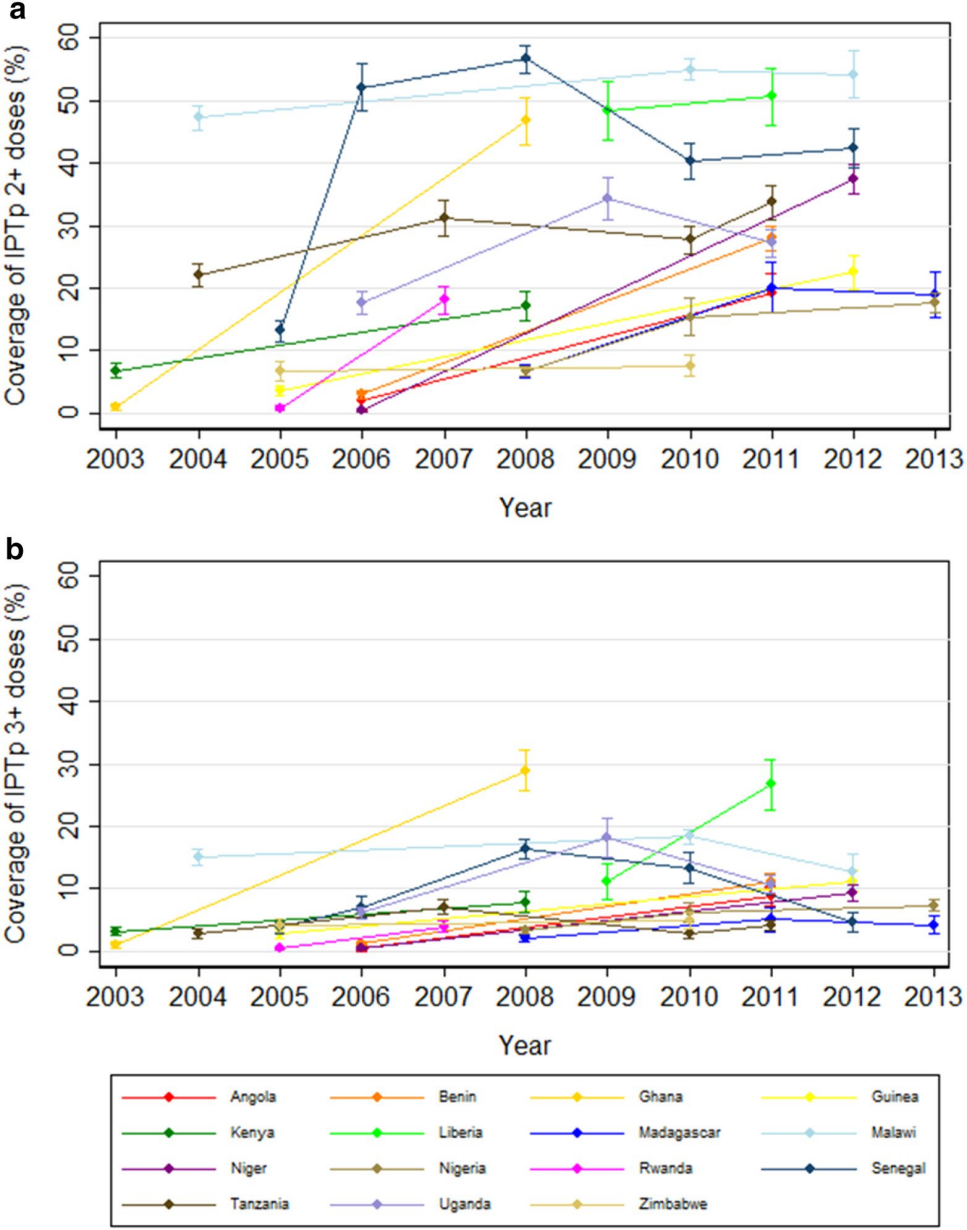
Coverage of IPTp-SP2+ (**a**) and IPTp3+ (**b**) among pregnant women by survey year, in countries with two or more available surveys. IPTp2+: Receipt of two or more doses of IPTp, IPTp3+: Receipt of three or more doses of IPTp. Improvement in coverage of IPTp-SP2+ (**a**) and IPTp-SP3+ (**b**) over time by country, for countries with multiple available surveys. The coverage of 2+ doses of IPTp is markedly higher than the coverage of 3+ doses, but both general improved over time between 2003 and 2013. Reflecting this, the median 5-year average annualized difference in coverage of IPTp-SP2+ was 12.4 % (IQR 3.8–28.0 %) and 5.5 % (IQR 0.8–8.4 %) for IPTp-SP3+

The coverage of three or more doses of IPTp-SP (IPTp-SP3+) was a median of 0.5, 6.1, and 9.9 % for the pre-, early-post, and late-post periods, respectively. For the late post-adoption period, the range was from 1.1 % (95 % CI 0.4–1.7 %) in Gabon in 2012 to 26.7 % (95 % CI 22.6–30.7 %) in Liberia in 2011, although the highest observed coverage of IPTp-SP3+ was 43.1 % (95 % CI 40.2–46.0 %) in Zambia in 2007, in the early post-implementation period (this is the only available survey for Zambia). To better examine these trends, coverage among countries with two or more available surveys was assessed. This analysis shows some fluctuations in coverage over time, but an overall improvement (Fig. 2). The median 5 year average annualized difference in coverage of IPTp-SP2+ was 12.4 percentage points (IQR 3.8–28.0 %), and 5.5 percentage points (IQR 0.8–8.4 %) for IPTp-SP3+.

### Missed opportunities

**Fig. 3:**
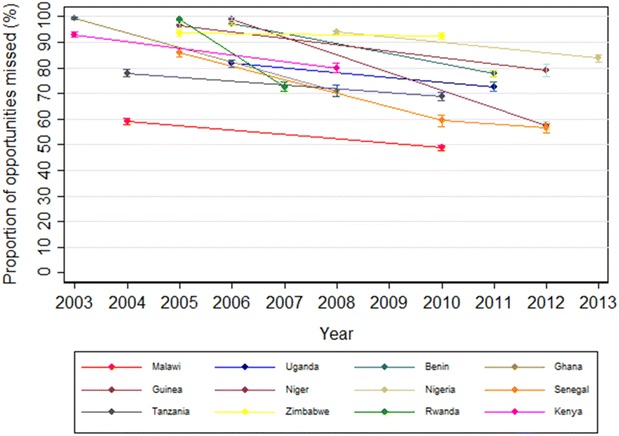
Proportion of missed opportunities to deliver IPTp-SP over time, by survey year, for all countries with two or more available surveys. Missed opportunities for IPTp delivery over time by country, showing countries for which there were at least two surveys. Overall, there was a median average decrease of 13.3 percentage points (IQR 7.5–33.6 %) per 5-year period

**Fig. 4:**
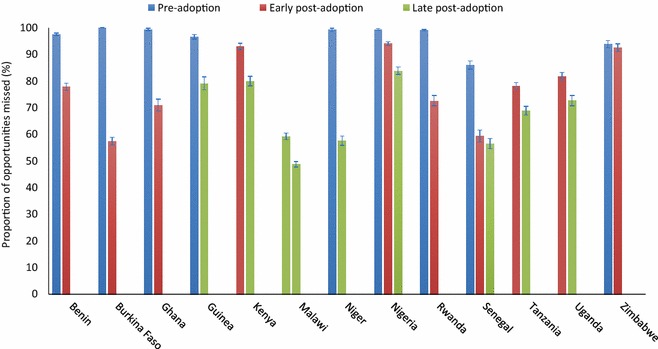
Missed opportunities for IPTp-SP delivery by country and IPTp policy adoption period for countries with two or more surveys. Missed opportunities for IPTp delivery by country and adoption period, showing countries for which there were data points in both the early and late post adoption periods. Overall, missed opportunities were seen in a median of 78.5 % of visits (IQR 69.3–91.1 %) in the early post adoption period and 71.6 % (IQR 56.8–78.9 %) in the late post adoption period

Using data from 172,506 women for whom both IPTp-SP and ANC data were available, a median of 99.0 % (IQR 96.5–99.2 %) of second and third trimester visits in pre-implementation surveys, 79.0 % (IQR 71.0–91.4 %) in early post-implementation surveys and 72.9 % (IQR 58.4–79.5 %) in late post-implementation surveys were missed opportunities for administration of IPTp-SP. The late post-implementation survey means ranged from 48.8 % (95 % CI 47.8–49.8 %) in Malawi in 2010 to 97.8 % (95 % CI 97.0–98.6 %) in Gabon in 2012. Across all time points, Zambia had the fewest missed opportunities: 44.3 % (95 % CI 42.6–46.1 %) in the early post-implementation period (no subsequent surveys were available for Zambia). Looking only at those countries where data from at least two time points were available shows that in individual countries there has been a reduction in missed opportunities over time, with a median 5 year average annualized difference of −13.0 percentage points (IQR −30.3 to −7.2 %) (Figs. 3, 4).

### Coverage of IPTp-SP2+ compared to TT2+

**Fig. 5:**
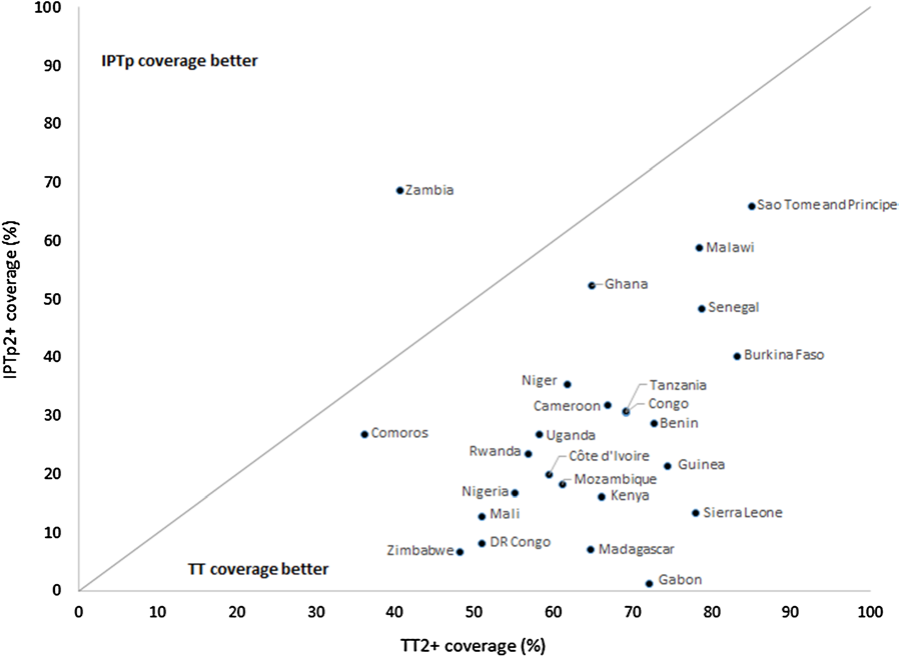
Comparison of coverage of two or more doses of IPTp-SP versus two or more doses of tetanus toxoid (TT) among primigravidae for countries with post-implementation data. IPTp2+: Receipt of two or more doses of IPTp, TT2+: Receipt of two or more doses of tetanus toxoid. Comparison of the coverage of IPTp2+ (on the *y*-axis) with TT2+ (on the *x*-axis) among primigravid women for each country with post-implementation survey data, using the latest available survey from each country. With the exception of Zambia, the delivery of TT2+ was notably better than that of IPTp2+ in all countries

IPTp-SP2+ and TT2+ coverage was available from 44 surveys, covering 45,808 women whose first pregnancy occurred in the 2 years preceding the survey. Median TT2+ coverage among primigravid women was 61.5 % (IQR 50.9–72.9 %), with a range from 31.6 % (95 % CIs 27.1–36.0 %) in Niger (2006) to 85.0 % (95 % CIs 79.0–91.0 %) in São Tomé and Príncipe (2008). Stratified by policy implementation period, the median TT2+ coverage in the pre-implementation surveys was 50.5 % (IQR 41.7–72.1 %), 61.2 % (IQR 51.0–72.7 %) in the early post-implementation surveys, and 68.0 % (IQR 60.0–73.8 %) in the late post-implementation surveys. Overall, coverage of TT2+ was markedly higher than IPTp-SP2+ coverage, with the exception of in Zambia, where coverage of IPTp-SP2+ exceeded that of TT2+, and Comoros, where IPTp-SP2+ and TT2+ coverage were approximately equal (Fig. 5). When this analysis was restricted to only women reporting attendance at two or more ANC visits, a similar pattern was seen (Additional file 2: Figure S1).

## Discussion

Despite high levels of ANC attendance, and demonstrated benefit of providing IPTp-SP, the coverage of IPTp-SP remains far below national and global targets (the President’s Malaria Initiative targets 85 % coverage, while Roll Back Malaria has called for 100 % coverage of at-risk populations), indicating that there are deficiencies in delivery of IPTp-SP at ANC. Although IPTp-SP coverage has generally increased over time, the change has been slow and much more progress is required to reach adequate coverage levels. This analysis clearly demonstrates that there are many missed opportunities to deliver IPTp-SP at ANC visits, far more so than for TT2+, highlighting the potential for improvements in service delivery at ANC to increase IPTp-SP coverage.

Although a recent meta-analysis finds that providers consistently state that women’s poor attendance at ANC is a significant contributing factor to the low coverage of IPTp-SP [14], the results from this study argue otherwise. Across all surveys, 76 % of women attend ANC three times, and 88 % attend twice, with the first visit on average occurring in the fourth month, yet in those same surveys only 19 % received two doses of IPTp-SP. Even in the late post-adoption period, seven or more years after a country formally adopted an IPTp-SP policy, a median of 72.9 % of ANC visits represented missed opportunities for IPTp-SP delivery. The reasons for these missed opportunities cannot be determined by this study; however, other studies suggest that healthcare worker confusion about the dose and timing, as well as inadequate supplies (including SP stock-outs, and lack of cups and drinking water) all play a role [14]. In addition, in some countries, the existence of restrictive policies recommending administration of IPTp-SP during specific weeks of gestation, or prohibiting its administration in the last 4 weeks of pregnancy certainly contributed to at least some of these missed opportunities prior to 2013 [21].

As these data include women who gave birth as many as 2 years preceding the survey, it may be difficult to see an increase in IPTp-SP in surveys conducted within 2 years of policy adoption. Therefore, coverage in both the early and late post-adoption periods was examined. Nonetheless, even when the data are restricted to only those surveys conducted seven or more years after policy adoption, coverage is still well below the universal coverage of preventive treatments recommended in the WHO Global Technical Strategy for Malaria 2016–2030 and called for in the Roll Back Malaria Action and Investment for Malaria 2016–2030 [22, 23]. Parasite resistance to SP in sub-Saharan Africa is becoming an increasing concern [24], and SP is no longer recommended for treatment of acute malaria. Whether this leads healthcare workers to not administer IPTp-SP due to concerns that it will no longer be effective remains unclear. A study in Ghana found that healthcare workers understand the distinction between treatment and chemoprophylaxis [25], suggesting that removing SP as a potential treatment should not have impacted administration of IPTp-SP as prophylaxis. However, research elsewhere found that women are prescribed SP for treatment, suggesting that not all healthcare workers appreciate this distinction [26], and still other research suggests that the recipients of the treatment also do not [27].

Zambia stands out as the only country with better coverage of IPTp-SP2+ than of TT2+. While the majority of countries until recently promoted a two dose policy for IPTp-SP, Zambia has always promoted a three dose policy; this, in conjunction with the excellent collaboration between the reproductive health department and the malaria control programme, has likely contributed to high two-dose coverage of IPTp [28]. Similarly, coverage of IPTp2+ is higher than average in Ghana where the policy is to provide three rather than two-doses of IPTp. In Ghana, a striking rise in coverage of IPTp2+, from 44 % in 2008 to 65 % in 2011 [29] was seen after conducting a nationwide campaign promoting this policy. These data suggest that a three dose policy may be associated with higher coverage levels. Hopefully, as more countries move to recommending IPTp-SP at each ANC visit, coverage of IPTp2+ will improve.

While the persistently low coverage of IPTp-SP is disheartening, the consistently higher delivery of TT suggests that IPTp-SP coverage can be improved. As TT is administered via injection and requires maintenance of the cold chain to preserve its effectiveness, it is seemingly a more complex intervention to deliver than IPTp-SP, thus it may at first seem surprising that delivery of TT2+ is consistently higher than that of IPTp-SP2+. However, IPTp is still relatively new compared to the more well-established TT. Although some countries adopted IPTp-SP as early as the 1990s, in most countries delivery of IPTp-SP was not a policy until 2004 [19]. In addition, IPTp-SP is recommended by malaria control programmes but implemented through reproductive healthcare; this may lead to less effective implementation. Unless there is excellent coordination between the malaria and reproductive programmes, discrepancies will exist in the guidance for IPTp-SP provided by the two programmes; this was highlighted in a recent review [21]. These discrepancies may cause healthcare worker confusion, resulting in failure to properly implement IPTp-SP [13]. Another possible contributor to the higher coverage of TT2+ than IPTp-SP2+ is the fact that TT can be given in the first trimester, while IPTp-SP can only be given starting in the second trimester. However, given the relatively small proportion of women who initiate ANC in the first trimester, this cannot fully explain the difference in coverage of the two interventions. Finally, differential recall bias may be contributing to the observed lower coverage of IPTp-SP compared to TT; women may be more likely to recall receiving an injection (a more painful experience) of TT than receiving pills for malaria prevention (which could even be mis-remembered as another intervention such as iron/folate supplementation), although this has not been studied. Further examination into the reasons for the discrepancy between delivery of IPTp-SP and TT and ways in which IPTp-SP treatment could benefit from the pre-existing structures of TT administration should be considered.

## Limitations

This analysis has several limitations. In these surveys, information on ANC attendance and receipt of IPTp-SP was obtained for pregnancies over the 5 years preceding the survey based only on women’s recall. The potential effect of recall bias was minimized by limiting analysis to the pregnancies of births in the 2 years preceding the survey. Also, although a large number of surveys are available across African countries during the time period covered by this analysis, for individual countries there may have been several years between surveys, making time trends difficult to assess. This was accounted for by examining changes across countries in five-year intervals since IPTp-SP policy adoption. Surveys were available for only a small number of countries in the last 2 years, so recent changes in IPTp-SP coverage could not be seen for many countries.

SP is contra-indicated in women who are HIV-positive and taking daily cotrimoxazole prophylaxis [24], but no data were available from these surveys to adjust for this. While in countries with high HIV prevalence, taking this into account may result in a significant decrease in missed opportunities, for most countries included in this analysis, the prevalence of HIV remains low and not accounting for women taking cotrimoxazole prophylaxis is unlikely to substantially affect the estimates of missed opportunities. Specifically, the median prevalence of HIV in 2008 (the median year of surveys included) in the countries in this analysis was 2.0 % [30], and the use of prophylactic cotrimoxazole was even lower. Therefore, the effect on calculating missed opportunities for administration of IPTp-SP would appear to be minimal in most countries. This is not controlled for in the analysis, but future work could take into account the prevalence of HIV and use of cotrimoxazole and aim to adjust the expected level of IPTp-SP coverage given HIV levels.

The results of this analysis could be affected by how timing of ANC visits was addressed. In the surveys, only the timing of the first ANC visit was available. For pregnancies in which the first visit reportedly occurred in the first trimester, it was assumed that all subsequent visits occurred after the first trimester. If more than one visit occurred in the first trimester, this assumption could lead to an underestimate of opportunities to administer IPTp-SP and consequently an overestimate of missed opportunities. However, this appears to be unlikely as the vast majority of surveys (three-quarters) with available information showed that less than 20 % of women made any visit in the first trimester and all surveys indicated a mean gestational age at first ANC greater than 3 months. Similarly, it was assumed that all ANC visits occurred at least 1 month apart, as the timing of ANC visits, other than the first visit, were not available in the surveys. IPTp-SP doses should be administered at an interval of at least 1 month, meaning that subsequent ANC visits taking place less than 4 weeks apart should not be counted as missed opportunities (and doing so could lead to an overestimate). However, the magnitude of the impact of this limitation is likely small. Specifically, given that a median of less than 49 % of women attend more than three ANC visits and the median timing of first ANC visit is in the fourth month, it seems unlikely that a large fraction of those women who go on to receive two or three additional ANC visits would do so at intervals less than 4 weeks during the subsequent 6 months of their pregnancy.

In rare cases, women reported receiving more IPTp-SP doses than ANC visits. This may be recall error, or it may reflect that the IPTp-SP questions in the survey are not sufficiently explicit to elicit accurate responses, that women are reporting treatment received for malaria as IPTp-SP, or that they are reporting the three IPTp-SP pills (one dose) as three separate doses. It could also reflect that they are receiving IPTp-SP from pharmacies or shops outside of their ANC visits. Not only would this contribute to error in the estimates of missed opportunities, but might be cause for larger public health concern if the quality of IPTp-SP outside of ANC is questionable.

## Conclusions

Despite a decade of implementation, and demonstrated added benefit of IPTp even where women are sleeping under ITNs [31], IPTp-SP coverage levels remain unacceptably low. Although many factors are involved [32], this analysis of missed opportunities clearly demonstrates that a large proportion of women who attend ANC are not receiving IPTp-SP, and that if these missed opportunities for delivery were eliminated, coverage levels would be much closer to the global targets, leading to improved maternal and newborn health [31]. The much higher levels of TT coverage suggest that it is possible for ANC clinics to improve service delivery. It is important to further investigate the context-specific factors that are driving the differences in TT2+ coverage and IPTp-SP2+ coverage in order to better address the bottlenecks to providing IPTp-SP, as well as to develop and assess interventions to address these gaps and challenges. The missed opportunities indicator could be added as a standard indicator to reports of future surveys, as this provides a useful assessment of the efficacy of IPTp-SP delivery at ANC.

## Additional files

10.1186/s12936-015-1033-4 Surveys included in the analysis. This file is a complete table of surveys included in the analysis, detailing the year of IPTp policy adoption, the year and type of survey, and IPTp and TT coverage.
10.1186/s12936-015-1033-4 Comparison of coverage of two or more doses of IPTp-SP versus two or more doses of Tetanus Toxoid (TT) among primigravidae reporting at least two antenatal care visits for countries with post-implementation data. This is a figure showing the proportion of primigravid women who received at least two doses of TT on the x-axis and at least two doses of IPTp-SP on the y-axis, restricted to those women who attended at least two antenatal care visits.
