# Supplementary material for: Missed opportunities to deliver intermittent preventive treatment for malaria to pregnant women 2003–2013: a systematic analysis of 58 household surveys in sub-Saharan Africa
Source: Malar J. 2015 Dec 23;14:521. doi: 10.1186/s12936-015-1033-4 (PMC4690242; doi:10.1186/s12936-015-1033-4)
Supplement: Supplementary file 1 — 10.1186/s12936-015-1033-4 Surveys included in the analysis. This file is a complete table of surveys included in the analysis, detailing the year of IPTp policy adoption, the year and type of survey, and IPTp and TT coverage. [file 12936_2015_1033_MOESM1_ESM.docx]

**Table S1 Surveys included in the analysis**

| **Country** | **Year IPTp policy imple-mented (18)** | **Survey Year** | **Survey Type^a^** | **Timing^b^** | **IPTp2+^c^ Coverage** | **IPTp3+^d^ Coverage** | **ANC2+^e^ Coverage** | **Missed opportunities to deliver IPTp** | **TT2+^f^ Coverage** |
| --- | --- | --- | --- | --- | --- | --- | --- | --- | --- |
|  |  |  |  |  | **% (95% CI)** | **% (95% CI)** | **% (95% CI)** | **% (95% CI)** | **% (95% CI)** |
| Angola | 2005 | 2006 | MIS | PI | 2 (.4-3.7) | 0.4 (0-1) |  |  |  |
|  |  | 2011 | MIS | EPI | 19.3 (16.2-22.4) | 8.8 (7.3-10.4) |  |  |  |
| Benin | 2005 | 2006 | DHS | PI | 3 (2.4-3.6) | 1.2 (.8-1.5) | 83.5 (81.4-85.5) | 97.4 (97-97.9) | 72.1 (69-75.3) |
|  |  | 2011 | DHS | EPI | 28 (26.1-30) | 11.2 (10-12.5) | 86 (84.3-87.7) | 77.8 (76.4-79.1) | 72.7 (69.4-76.1) |
| Burkina Faso | 2005 | 2003 | DHS | PI | 0 (0-.1) | 0 (0-.1) | 62.9 (59.2-66.5) | 99.9 (99.8-100) | 49.6 (44.6-54.5) |
|  |  | 2010 | DHS | EPI | 39.5 (37.3-41.7) | 5.4 (4.5-6.2) | 92 (90.9-93.1) | 57.5 (56-58.9) | 83.2 (80.5-85.8) |
| Burundi | NONE | 2010 | DHS | PI | 0.3 (0-.6) | 0.3 (0-.6) | 95.5 (94.7-96.2) | 99.7 (99.4-100) | 48.3 (43.3-53.2) |
|  |  | 2012 | MIS | PI | 0.1 (0-.2) | 0 (0-.1) |  |  |  |
| Cameroon | 2004 | 2011 | DHS | LPI | 27 (24.8-29.1) | 12.2 (10.9-13.4) | 81.9 (78.8-85) | 76.1 (74.9-77.3) | 66.9 (63.3-70.5) |
| Comoros | 2003 | 2012 | DHS | LPI | 31.5 (27.8-35.3) | 11.3 (9.1-13.4) | 87.2 (84.8-89.7) | 73.2 (70.2-76.2) | 36.1 (27.4-44.8) |
| Congo | 2006 | 2011 | DHS | EPI | 35.3 (32-38.7) | 17.3 (14.8-19.8) | 91.9 (90.3-93.6) | 72.7 (70.8-74.6) | 69.2 (64-74.4) |
| Congo, DRC | 2004 | 2007 | DHS | EPI | 7 (5.5-8.5) | 2.6 (1.8-3.3) | 82 (78.5-85.6) | 91.4 (89.8-92.9) | 51 (43.4-58.6) |
| Côte d'Ivoire | 2005 | 2011 | DHS | EPI | 20.2 (17.3-23.2) | 8.2 (6.7-9.7) | 81.9 (79.6-84.1) | 79.6 (76.7-82.6) | 59.4 (53.1-65.8) |
| Ethiopia | NONE | 2005 | DHS | PI | 1.2 (.7-1.7) | 0.7 (.4-1) | 23.1 (20.4-25.7) | 98 (96.9-99) | 32.7 (28-37.5) |
| Gabon | 2003 | 2012 | DHS | LPI | 2.8 (1.8-3.7) | 1.1 (.4-1.7) | 93.8 (92.4-95.1) | 97.8 (97-98.6) | 72.1 (66.3-78) |
| Ghana | 2003 | 2003 | DHS | PI | 1.1 (.5-1.6) | 0.8 (.3-1.3) | 88 (85.8-90.2) | 99.2 (98.8-99.7) | 54.8 (48.9-60.7) |
|  |  | 2008 | DHS | EPI | 46.8 (43-50.6) | 28.9 (25.6-32.1) | 93 (91.3-94.6) | 71 (68.8-73.3) | 64.9 (58-71.7) |
| Guinea | 2005 | 2005 | DHS | PI | 3.6 (2.8-4.5) | 2.8 (2.1-3.5) | 76.3 (72.3-80.4) | 96.5 (95.7-97.4) | 73.1 (68.1-78.1) |
|  |  | 2012 | DHS | LPI | 22.5 (19.9-25.2) | 11.1 (9.4-12.9) | 82.5 (79.6-85.3) | 79.1 (76.7-81.5) | 74.5 (69.6-79.3) |
| Kenya | 1999 | 2003 | DHS | EPI | 6.8 (5.7-8) | 3.1 (2.4-3.8) | 84.3 (82.5-86.1) | 93 (91.9-94) | 59.6 (55.4-63.7) |
|  |  | 2008 | DHS | LPI | 17.1 (14.8-19.5) | 7.8 (6.2-9.5) | 88.4 (86.2-90.5) | 79.9 (78.1-81.8) | 66.1 (61.1-71.2) |
| Liberia | 2004 | 2009 | MIS | EPI | 48.5 (43.8-53.1) | 11.1 (8.1-14.1) |  |  |  |
|  |  | 2011 | MIS | LPI | 50.7 (46.1-55.3) | 26.7 (22.6-30.7) |  |  |  |
| Madagascar | 2004 | 2008 | DHS | EPI | 6.8 (5.6-7.9) | 1.9 (1.4-2.5) | 85.4 (83.5-87.2) | 92.9 (91.8-94) | 64.7 (60.6-68.9) |
|  |  | 2011 | MIS | LPI | 20.1 (16.2-24.1) | 5 (3.1-6.9) |  |  |  |
|  |  | 2013 | MIS | LPI | 18.9 (15.2-22.6) | 4.2 (2.7-5.6) |  |  |  |
| Malawi | 1993 | 2004 | DHS | LPI | 47.2 (45.4-49.1) | 15 (13.7-16.4) | 92.6 (91.5-93.7) | 59.2 (58-60.5) | 73.2 (69.8-76.5) |
|  |  | 2010 | DHS | LPI | 55 (53.4-56.6) | 18.3 (17.1-19.5) | 95.4 (94.8-96.1) | 48.8 (47.8-49.8) | 78.4 (75.8-81) |
|  |  | 2012 | MIS | LPI | 54.2 (50.4-57.9) | 12.8 (10-15.6) |  |  |  |
| Mali | 2003 | 2006 | DHS | EPI | 11.3 (9.7-12.9) | 5.9 (4.7-7) | 65.8 (62.5-69) | 86.3 (84.6-88) | 51 (46.7-55.3) |
| Mozambique | 2006 | 2011 | DHS | EPI | 19.9 (17.8-21.9) | 9.8 (8.2-11.3) | 85.6 (83.3-87.9) | 79 (77.2-80.8) | 61.2 (57.2-65.1) |
| Namibia | 2005 | 2006 | DHS | PI | 11.6 (9.7-13.5) | 7.7 (6.1-9.3) | 93.2 (91.6-94.8) | 89.9 (88.2-91.5) | 38.6 (34.1-43.2) |
| Niger | 2005 | 2006 | DHS | PI | 0.5 (.2-.8) | 0.4 (.1-.7) | 42.2 (38.1-46.2) | 99.1 (98.5-99.7) | 31.6 (27.1-36) |
|  |  | 2012 | DHS | LPI | 37.5 (35.1-39.8) | 9.2 (7.9-10.6) | 81 (78.9-83.2) | 57.6 (55.8-59.3) | 61.7 (57.6-65.9) |
| Nigeria | 2004 | 2003 | DHS | PI | 1.1 (.5-1.7) | 0.5 (.2-.9) | 60.1 (55-65.2) | 99.2 (98.9-99.6) | 41.7 (35.5-48) |
|  |  | 2008 | DHS | EPI | 6.7 (6-7.5) | 3.3 (2.8-3.7) | 58 (55-60.9) | 94 (93.3-94.6) | 50.9 (47.6-54.2) |
|  |  | 2010 | MIS | EPI | 15.4 (12.5-18.4) | 6.1 (4.5-7.8) |  |  |  |
|  |  | 2013 | DHS | LPI | 17.7 (16.1-19.3) | 7.2 (6.3-8.2) | 64 (61-66.9) | 83.8 (82.4-85.2) | 55.1 (51.9-58.3) |
| Rwanda | 2005-2008 | 2005 | DHS | PI | 0.9 (.5-1.2) | 0.5 (.3-.8) | 81.9 (80.1-83.6) | 99 (98.6-99.3) | 50.5 (46.5-54.5) |
|  |  | 2007 | DHS | EPI | 18.1 (15.9-20.4) | 3.9 (2.9-4.8) | 91.2 (89.6-92.8) | 72.6 (70.7-74.5) | 56.8 (51.1-62.5) |
| Sao Tome and Principe | 2004 | 2008 | DHS | EPI | 64.6 (59.5-69.6) | 5.1 (2.1-8) | 93.2 (90.4-96) | 62.3 (59.8-64.8) | 85 (79-91) |
| Senegal | 2004 | 2005 | DHS | PI | 13.2 (11.4-14.9) | 3.7 (2.8-4.7) | 88.9 (87.1-90.8) | 86 (84.4-87.5) | 76 (72.8-79.1) |
|  |  | 2006 | MIS | EPI | 52.1 (48.3-56) | 7 (5.4-8.7) |  |  |  |
|  |  | 2008 | MIS | EPI | 56.6 (54.4-58.9) | 16.4 (14.8-18) |  |  |  |
|  |  | 2010 | DHS | EPI | 40.3 (37.4-43.2) | 13.2 (10.7-15.7) | 91.1 (89.7-92.4) | 59.4 (57.2-61.6) | 68.3 (64.5-72) |
|  |  | 2012 | DHS | LPI | 42.5 (39.3-45.6) | 4.5 (3-6) | 89.6 (87.6-91.6) | 56.5 (54.6-58.4) | 78.8 (73.5-84) |
| Sierra Leone | 2004 | 2008 | DHS | EPI | 12.9 (10.7-15.2) | 5.4 (4.2-6.6) | 90.6 (88.1-93) | 91.3 (89.8-92.8) | 78 (72.9-83.2) |
| Swaziland | NONE | 2006 | DHS | PI | 0 (0-0) | 0 (0-0) | 96.1 (95-97.3) | 100 (100-100) | 73.7 (68.6-78.8) |
| Tanzania, United Republic of | 2001 | 2004 | DHS | EPI | 22.1 (20.3-24) | 2.8 (2.1-3.4) | 94.9 (93.6-96.1) | 78 (76.7-79.4) | 73.5 (69.6-77.4) |
|  |  | 2007 | AIS | EPI | 31.3 (28.4-34.1) | 7 (5.8-8.3) |  |  |  |
|  |  | 2010 | DHS | LPI | 27.7 (25.4-30) | 2.8 (2.1-3.4) | 93.6 (92.4-94.8) | 68.9 (67.2-70.5) | 69.2 (64-74.3) |
|  |  | 2011 | AIS | LPI | 33.8 (31-36.5) | 4.1 (3.2-4.9) |  |  |  |
| Uganda | 2000 | 2006 | DHS | EPI | 17.6 (15.7-19.5) | 6.1 (5.1-7.1) | 89.2 (87.7-90.7) | 81.7 (80.2-83.2) | 56.5 (51.6-61.4) |
|  |  | 2009 | MIS | LPI | 34.4 (31-37.7) | 18 (14.9-21.2) |  |  |  |
|  |  | 2011 | DHS | LPI | 27.3 (25.1-29.5) | 10.6 (9.2-12.1) | 91.3 (89.8-92.7) | 72.7 (70.8-74.5) | 58.2 (53.7-62.7) |
| Zambia | 2001 | 2007 | DHS | EPI | 65.9 (63.2-68.6) | 43.1 (40.2-46) | 94.6 (93.5-95.6) | 44.3 (42.6-46.1) | 40.7 (35.9-45.5) |
| Zimbabwe | 2004 | 2005 | DHS | PI | 6.7 (5.2-8.2) | 4 (3-5.1) | 91.5 (90-93) | 93.8 (92.5-95.1) | 60.9 (56.8-65) |
|  |  | 2010 | DHS | EPI | 7.7 (6-9.3) | 4.9 (3.6-6.1) | 84.3 (81.9-86.7) | 92.5 (91.1-93.8) | 48.3 (43.7-52.8) |

^a^ DHS: Demographic and Health Survey, MIS: Malaria Indicator Survey, AIS: AIDS Indicator Survey

^b^ Timing with respect to implementation: PI: Pre-implementation, EPI: Early post-implementation, LPI: Late post-implementation

^c^ IPTp2+: Receipt of 2 or more doses of IPTp

^d^ IPTp3+: Receipt of 3 or more doses of IPTp

^e^ ANC2+: Attendance at 2 or more antenatal care visits

^f^ TT2+: Receipt of 2 or more doses of tetanus toxoid
