# Supplementary material for: Missed opportunities to deliver intermittent preventive treatment for malaria to pregnant women 2003–2013: a systematic analysis of 58 household surveys in sub-Saharan Africa
Source: Malar J. 2015 Dec 23;14:521. doi: 10.1186/s12936-015-1033-4 (PMC4690242; doi:10.1186/s12936-015-1033-4)
Supplement: Supplementary file 2 — 10.1186/s12936-015-1033-4 Comparison of coverage of two or more doses of IPTp-SP versus two or more doses of Tetanus Toxoid (TT) among primigravidae reporting at least two antenatal care visits for countries with post-implementation data. This is a figure showing the proportion of primigravid women who received at least two doses of TT on the x-axis and at least two doses of IPTp-SP on the y-axis, restricted to those women who attended at least two antenatal care visits. [file 12936_2015_1033_MOESM2_ESM.pdf]

Figure S1 Comparison of coverage of two or more doses of IPTp-SP versus two or more doses of Tetanus Toxoid (TT) among primigravidae reporting at least two antenatal care visits for countries with post-implementation data

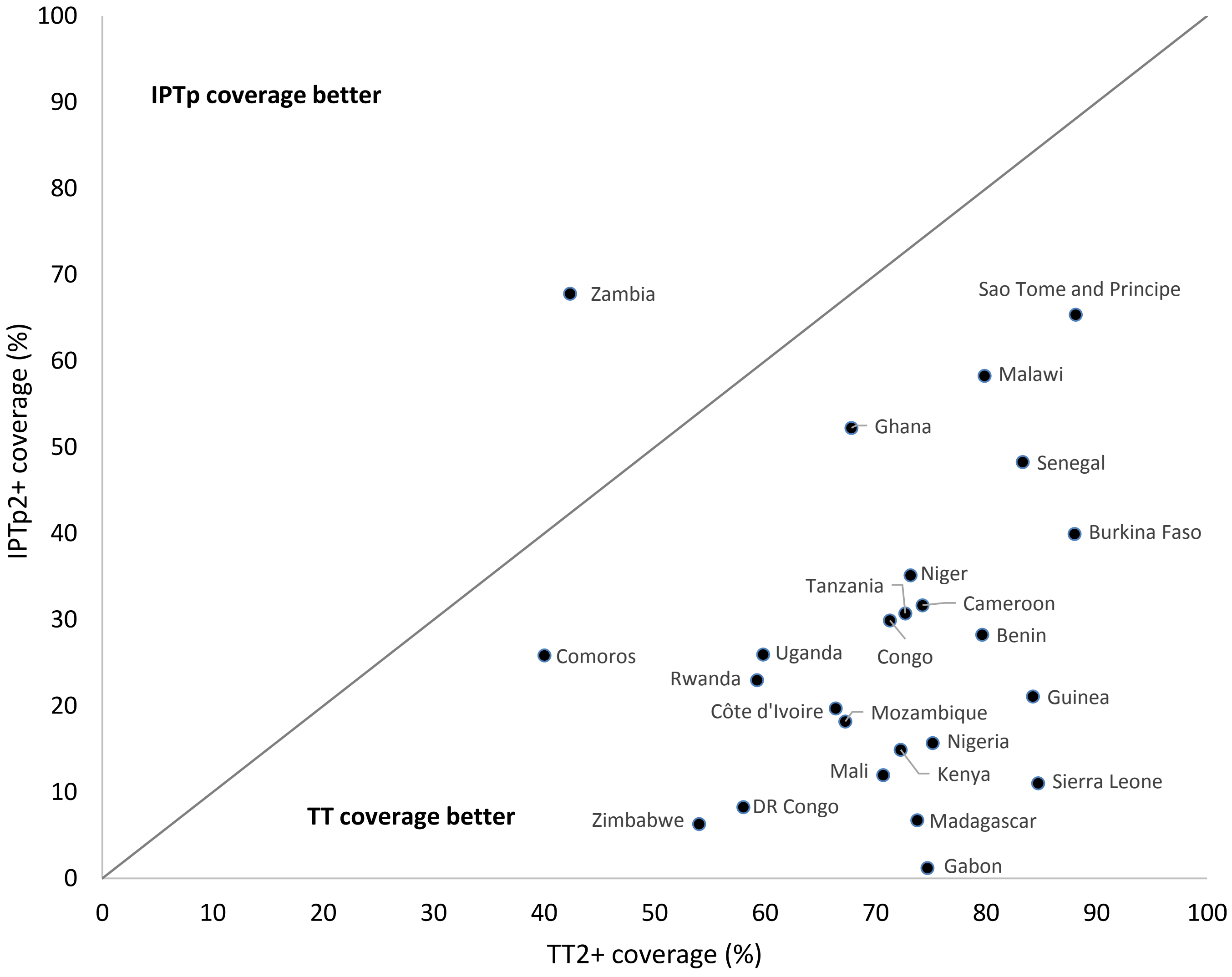

IPTp2+: Receipt of 2 or more doses of IPTp; TT2+: Receipt of 2 or more doses of tetanus toxoid

Comparison of the coverage of IPTp2+ (on the y-axis) with TT2+ (on the x-axis) among primigravid women who attended at least two antenatal care visits, for each country with post-implementation survey data, using the latest available survey from each country. With the exception of Zambia, the delivery of TT2+ was notably better than that of IPTp2+ in all countries.
